# Relationship Between Amyloid-β Deposition and Blood–Brain Barrier Dysfunction in Alzheimer’s Disease

**DOI:** 10.3389/fncel.2021.695479

**Published:** 2021-07-19

**Authors:** Dong Wang, Fanglian Chen, Zhaoli Han, Zhenyu Yin, Xintong Ge, Ping Lei

**Affiliations:** ^1^Department of Geriatrics, Tianjin Medical University General Hospital, Tianjin, China; ^2^Tianjin Geriatrics Institute, Tianjin, China; ^3^Tianjin Neurological Institute, Tianjin, China; ^4^Department of Neurosurgery, Tianjin Medical University General Hospital, Tianjin, China

**Keywords:** amyloid-β, blood–brain barrier, Alzheimer’s disease, low-density lipoprotein receptor-related protein 1, receptor for advanced glycation end products, P-glycoprotein

## Abstract

Amyloid-β (Aβ) is the predominant pathologic protein in Alzheimer’s disease (AD). The production and deposition of Aβ are important factors affecting AD progression and prognosis. The deposition of neurotoxic Aβ contributes to damage of the blood–brain barrier. However, the BBB is also crucial in maintaining the normal metabolism of Aβ, and dysfunction of the BBB aggravates Aβ deposition. This review characterizes Aβ deposition and BBB damage in AD, summarizes their interactions, and details their respective mechanisms.

## Introduction

Alzheimer’s disease (AD) accounts for 60–70% of all dementia cases. There are 50 million AD patients worldwide and 10 million new cases are reported annually (Alzheimer’s Association, 2021). AD is a neurodegenerative disease characterized by brain lesions related to a variety of cellular and molecular changes. The primary manifestations of AD are a progressive decline in memory, cognition, thinking, behavior, and daily activities (van Dyck, 2018). There are two main forms: sporadic AD (SAD), which accounts for 95% of cases; and familial AD (FAD), which accounts for 5% of cases (Thal and Fandrich, 2015). The cause of early-onset FAD is linked to aberrant alleles. However, the full etiology of most AD cases remains unclear (Oikari et al., 2020). There are several hypotheses considering the pathogenesis of AD, including those emphasizing roles for amyloid-β (Aβ), the Tau protein, oxidative stress and calcium, glutamatergic neurotransmission, and acetylcholine (Bao et al., 2012; Toga et al., 2016; Sanabria-Castro et al., 2017).

**FIGURE 1:**
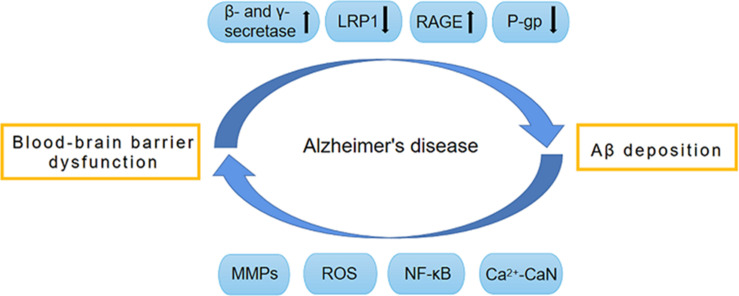
Vicious circle formed by BBB dysfunction and Aβ deposition. The dysfunction of BBB promotes the production of Aβ by activating β-secretase and γ-secretase. Meanwhile, BBB dysfunction also impairs the normal transport of Aβ, and leads to the abnormal accumulation of Aβ. The normal transport of Aβ through BBB depends on a variety of transporters on BBB, such as LRP1, RAGE and P-gp. During the development of AD, these transporters have abnormal expression and function, which causes abnormal Aβ transport and deposition, thereby leading to BBB destruction. The pathological factors involved in this procedure include MMPs, ROS, NF-κB, and Ca2 + -CaN. AD, Alzheimer’s disease; Aβ, Amyloid-β; BBB, Blood–brain barrier; CaN, Calcineurin; RAGE, receptor for advanced glycation end products; LRP1, low-density lipoprotein receptor-related protein 1; MMPs, Metalloproteinases; NF-κB, Nuclear factor-κB; P-gp, P-glycoprotein; ROS, reactive oxygen species.

The amyloid-cascade hypothesis (ACH) states that Aβ accumulation and deposition in the brain is the key initial step in AD pathogenesis. Due to the genetic and pathologic links between Aβ and AD, this theory has been widely acknowledged and has been a dominant driver of active investigation over the past three decades (Tcw and Goate, 2017; He J.T. et al., 2020). Aβ is composed of 36–46 amino acids and has neurotoxic effects that impair the blood–brain barrier (BBB). Evidence suggests that the etiology of AD may be related to the dysfunction of Aβ clearance from the brain (Wang et al., 2006). However, the exact mechanisms of Aβ accumulation and resultant BBB damage are poorly understood (Bourassa et al., 2019). The presence of multiple Aβ clearance mechanisms in the brain reduces harmful effects of Aβ and the most momentous is Aβ transfer across the BBB (Tanzi et al., 2004). This essential structure controls the exchange of substances between the brain and the blood, maintaining homeostasis of the central nervous system (CNS). It is a complex, dynamic, and adaptable interface, rather than a simple physical barrier (Zlokovic, 2008). BBB dysfunction and Aβ deposition may lead to a vicious cycle that causes AD development (Figure 1). Only five drugs have so far been approved by the US Food and Drug Administration (FDA) for the improvement of AD symptoms, all of which have serious side effects (van der Kant et al., 2020). Effective and safe regimens for curing or changing the course of AD are not currently available (Goossens et al., 2017).

## The Blood–Brain Barrier and Alzheimer’s Disease

The BBB is a dynamic biological and physical barrier between the peripheral circulation and the CNS. It separates the blood from the brain, providing a stable and optimal environment to maintain the normal functioning of neurons, transporters, and ion channels that are expressed on the BBB and involved in ion balance and synaptic function (Chakraborty et al., 2017).

**FIGURE 2:**
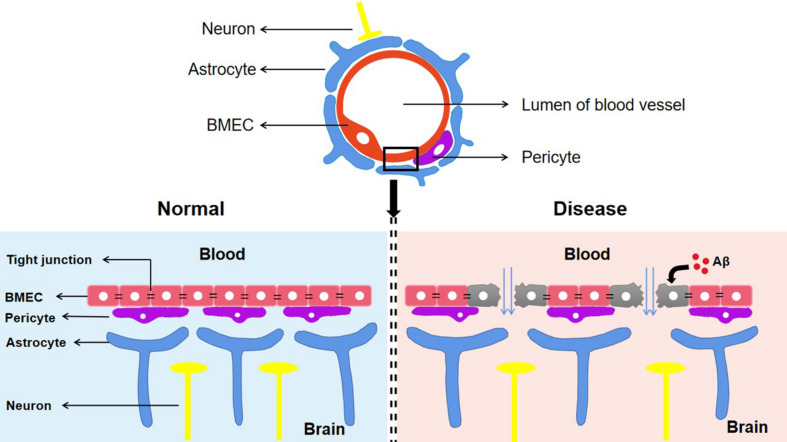
The components of blood–brain barrier and its manifestations in different states. The BBB composed of astrocytes, pericytes and BMEC is an indispensable structure that mediates the material exchange between blood and brain. TJs, the highly specialized intercellular adhesion complex, acts as a selective barrier to regulate the transport of non-ionic molecules between blood and brain. BBB contributes to maintaining a stable microenvironment in the brain and normal function of neurons. In Alzheimer’s disease, cytotoxic Aβ destroys BMEC and TJs associated proteins, which suggests the destruction of the TJ system and the loss of BBB integrity. Aβ, Amyloid-β; BBB, Blood–brain barrier; TJs, Tight junctions; BMEC, brain microvascular endothelial cells.

The BBB is composed of astrocytes, pericytes, and brain microvascular endothelial cells (BMECs) (Huang Z. et al., 2020). Among these, BMECs express a wide range of transporters and receptors involved in the selective uptake of substances from the blood to the brain (Georgieva et al., 2020). Tight junctions (TJs), which are highly specialized intercellular-adhesion complexes, exist in epithelial and endothelial cells (Zhao et al., 2021). The TJs in BMECs act as selective barriers to regulate the movement of non-ionic molecules between the blood and brain through the paracellular pathway to maintain cerebral homeostasis (Huang Z. et al., 2020). Numerous membrane proteins have been identified in TJ complexes including the following: occludins; the claudins CLDN-1, CLDN-3, CLDN-5, and CLDN-12; the cytoplasmic-attachment zonula proteins ZO-1, ZO-2, and ZO-3; junctional adhesion molecules (JAMs); and tricellulin (Cuevas et al., 2019). In addition, numerous studies have found that the integrity of the BBB is closely related to the functional state of TJs. The abnormal expression or distribution of TJ-related proteins leads to the impairment of TJ integrity and increases the permeability of the BBB, which is related to a variety of CNS diseases including AD, stroke, and subarachnoid hemorrhages (SAHs) (Yamazaki et al., 2019). Cytotoxic Aβ destroys BMECs and TJ-associated proteins, resulting in loss of BBB integrity (Yamazaki et al., 2019; Figure 2). In a recent study, TJ destruction, incremental BBB permeability, and decreased expression levels of CLDN-1 and CLDN-5 were observed in AD patients (Yamazaki et al., 2019). These findings indicate that Aβ is the initial cause of disruption of TJ and BBB integrity. Some studies also suggest that impairments of blood vessels could induce BBB dysfunction and cerebral hypo-perfusion, which are associated with following Aβ accumulation and neuronal injury (Sweeney et al., 2018; He J.T. et al., 2020). Moreover, the BBB breakdown caused by damage to blood vessels is more likely to manifest as cerebral microbleeds (microhemorrhages) in people who have increased genetic risk of AD (Sweeney et al., 2018).

Decades of evidence have established the critical role of the BBB in AD (Montagne et al., 2017; van de Haar et al., 2017; Rosas-Hernandez et al., 2020). Previous studies showed that the breakdown of the BBB reduced Aβ clearance and caused Aβ deposition by inducing inflammation, increasing AD risk (Huang Z. et al., 2020). Dysfunction of the BBB includes its disruption and abnormal expression of Aβ transporters (Bourassa et al., 2019).

Previous work showed elevated levels of albumin in the cerebrospinal fluid (CSF) and serum of AD patients. Albumin in the CSF is derived from the peripheral circulation, indicating that the BBB integrity in AD patients is damaged and leakage occurs (Bowman et al., 2007). Another study used immunocytochemical techniques to detect serum- and plasma-derived molecules in the brain and found that hemoglobin-derived peptides and prothrombin levels were elevated in AD patients. Since the healthy brain does not produce or secrete these substances, their presence was considered to reflect BBB destruction (Zipser et al., 2007). Morphological changes, such as basal membrane thickening and decreased micro-vessel density, which have been observed post-mortem in the brains of AD patients and in animal models, are indicative of BBB damage (Lepelletier et al., 2017; Thomsen et al., 2017). These experimental results demonstrate the destruction of the BBB in AD.

Various Aβ transporters situated on the BBB regulate the influx and efflux of Aβ. The expression of these transporters is altered in AD. Among these, low-density lipoprotein receptor-related protein 1 (LRP1) and P-glycoprotein (P-gP) transporters control the decrease of Aβ efflux, while the receptor for advanced glycation end-products (RAGE) transporter controls the increase of Aβ influx. Their influence on Aβ deposition and corresponding mechanisms are discussed in the following sections. Abnormal transporter function on the BBB has been identified in other pathological conditions, such as stroke, inflammation, obesity, and diabetes, and these are also considered as risk factors for AD.

## Amyloid-β

Research on AD often focuses on the role of Aβ due to the pathologic and genetic associations between them (Selkoe and Hardy, 2016; Tcw and Goate, 2017). Extensive evidence indicates that Aβ removal plays a more pivotal role in the process of Aβ accumulation in the brains of AD patients than does an increase in Aβ production (Zlokovic, 2011). The general consensus – the ACH – indicates that effective Aβ clearance is critical to maintain normal neurological function; failure of this process may result in the accumulation of Aβ, initiating neurodegeneration and cognitive impairment (He J.T. et al., 2020; Iraji et al., 2020).

Amyloid-β is a produced by the sequential cleavage of the Aβ precursor protein (AβPP) expressed in neurons, brain endothelial cells, and astrocytes by β-secretase 1 (BACE1) and γ-secretase (Menendez-Gonzalez et al., 2018; Saretz et al., 2021). The AβPP is a long, insoluble amyloid fiber and single-pass transmembrane protein (Chakravarthy et al., 2017). Mutations within or flanking the Aβ domain of AβPP are associated with early-onset autosomal dominant forms of FAD (Johnson et al., 2017). In addition, Aβ produced peripherally by various cell types is transported into the brain across the BBB through transcytosis mediated by receptors such as RAGE (Greenberg et al., 2020).

Amyloid-β peptide fragments vary in length from around 36 to 46 amino acids. Those most commonly found in senile plaques are the 40 amino acid (Aβ_1__–__40_) and 42 amino acid (Aβ_1__–__42_) isoforms (Gireud-Goss et al., 2020). Aβ_1__–__40_ is soluble, has low toxicity, and is commonly found in healthy brains, accounting for 90% of the total Aβ. In contrast, Aβ_1__–__42_ is highly neurotoxic, is mainly found in AD brain tissue, and accounts for less than 10% of the total Aβ (Kumar et al., 2018; Sharda et al., 2021).

Several forms of Aβ have been found in the brains of patients with AD, including monomers, oligomers, and fibrils. Debate remains as to which conformation has the highest toxicity. Some studies have suggested that Aβ_1__–__40_ is the most toxic and aggregation-prone isoform (Urbanc et al., 2002), It has also been reported that extracellular plaques are composed of fibrillary Aβ deposits and are associated with neurotoxicity (Moreno et al., 2009). However, there is no clear correlation between the number of neurons and amyloid deposition (Moreno et al., 2009), More recent work showed that Aβ-related synaptotoxicity was associated with the accumulation of Aβ oligomers (Seixas et al., 2017). The oligomer hypothesis suggests that soluble Aβ oligomers reduce cell survival and damage synapses, mediating memory loss (Amar et al., 2017). In an AD animal model, synaptic defects and cognitive impairment were reversed by reducing soluble Aβ expression levels (Al et al., 2020). There is increasing evidence that the accumulation of soluble Aβ oligomers, rather than mature amyloid fibrils, is the earliest pathogenic event in AD (Bilousova et al., 2016). Therefore, the level of soluble Aβ is considered to be a strong predictor of synaptic dysfunction, cognitive impairment, and neuropathologies in the brains of AD patients (Koss et al., 2016; Ono, 2018). However, although small Aβ oligomers are considered to be toxic, the exact properties of these transient heterogeneous aggregates remain unclear (Soto-Rojas et al., 2021a).

The accumulation of Aβ triggers neurofibrillary tangles, oxidative stress, microglial activation, synaptic dysfunction, synaptic loss, and the inflammatory response. Increasing Aβ peptides from high-nanomolar to low-micromolar concentrations inhibits synaptic function, which is associated with neurotoxicity and neuron loss. High concentrations of Aβ disrupt neurotransmission at a postsynaptic level (Gulisano et al., 2019b; Gireud-Goss et al., 2020). By contrast, high-nanomolar concentrations of Aβ impact the endocytosis of synaptic vesicles (SVs) at presynaptic sites, which results in inhibitory effects (Park et al., 2013). Increasing the concentration of Aβ causes synaptic depression and may lead to synaptic loss in AD (Gulisano et al., 2019a; Ortiz-Sanz et al., 2020). Soluble Aβ oligomers are thought to disrupt learning and memory because they block the long-term potentiation (LTP) of the hippocampus, which is associated with these functions (Chakravarthy et al., 2017; Kasza et al., 2017). Hippocampal LTP is a synaptic model of memory, particularly long-term memory, in the brain (Zhang et al., 2020). LTP is often used as an electrophysiological correlation between learning and memory when studying synaptic function (Kent et al., 2020). Many studies suggest that low (picomolar) concentrations of Aβ enhance LTP, while high (nanomolar) concentrations inhibit LTP (Gulisano et al., 2019b; He et al., 2019). A recent study reported neuroprotective effects of arginine vasopressin (AVP) on Aβ-induced impairments of memory behavior and LTP (Zhang et al., 2020). In addition, sleep disturbance is considered to be an early sign of AD because Aβ deposition can alter sleep architecture (He C. et al., 2020). Sleep can promote the clearance of Aβ; thus, the presence of a sleep disorder will increase the level of Aβ (Holth et al., 2019).

Amyloid plaques formed by Aβ aggregation are considered to be a pathological trigger of AD (Uddin et al., 2020). They alter the shapes of neurons, increase the distance between them, and eventually prevent interneuron communication. These processes occur in an area of the brain related to memory and cognition, leading to impairments of these abilities in AD patients (Selkoe and Hardy, 2016; Kumar et al., 2018). In addition, recent studies have shown that both Aβ plaques and oligomers have strong toxic effects on synapses, which block the function of proteasomes, alter intracellular Ca^2+^ levels, and promote inflammation (Al et al., 2020).

**FIGURE 3:**
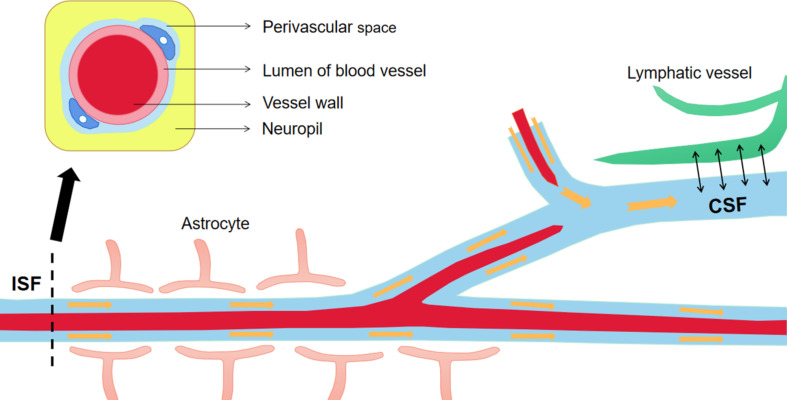
The material exchanges between perivascular space, ISF and CSF. The perivascular space is a normal anatomical structure in the nervous system, which has important physical and immune functions. Continuous brain ISF enters into CSF through perivascular space (Virchow-Robin spaces, VRS), and drains into the blood through lymphatic vessels. This is one of the pathways to clear Aβ from the brain. Aβ, Amyloid-β; ISF, interstitial fluid; CSF, cerebrospinal fluid.

**FIGURE 4:**
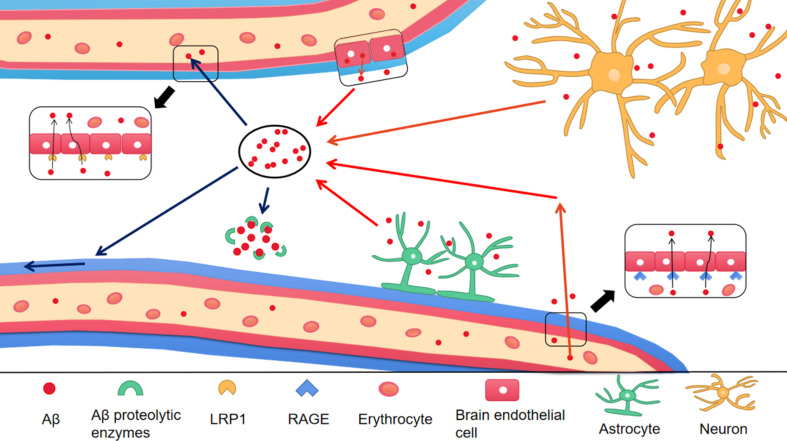
The production and clearance of Aβ in the brain. The red arrow represents the source of Aβ in the brain, and the blue arrow represents the ways of Aβ clearance. Different type of cells in the brain take part in the production of Aβ, including astrocytes, neurons and endothelial cells. In addition, Aβ in peripheral blood enters into the brain through RAGE, a transporter on BBB. There are a variety of Aβ clearance ways. (1) Aβ enters into peripheral blood through LRP1 transporter on BBB; (2) Aβ enters into CSF through perivascular space; (3) Aβ is degraded by Aβ proteolytic enzymes. Aβ, Amyloid-β; BBB, Blood–brain barrier; LRP1, low-density lipoprotein receptor-related protein 1; RAGE, receptor for advanced glycation end products.

To prevent Aβ deposition and circumvent the toxic effects, various Aβ-scavenger pathways work together in the brain, including BBB transportation, extracellular degradation by Aβ-proteolytic enzymes, cellular uptake, intracellular degradation, interstitial fluid (ISF) bulk flow, and CSF absorption (Ma et al., 2018; Abdallah et al., 2021). Studies have shown that 50% of Aβ is transported into the blood across the BBB by LRP-mediated transcytosis and degradation of Aβ in vascular smooth-muscle cells (Tanzi et al., 2004). Enzymatic degradation can be extracellular or intracellular. The extracellular degradation of Aβ mainly depends on protein-degrading enzymes secreted by cells, including neprilysin, insulin-degrading enzymes, and endothelin-converting enzymes. Aβ can also be absorbed by neurons, microglia, and astrocytes, as well as being degraded by proteases and lysosomes (Farris et al., 2007; Tarasoff-Conway et al., 2015). A continuous and slow flow of brain ISF (which surrounds neurons) into the CSF (which surrounds the brain), followed by drainage into the blood across the perivascular space, accounts for 10–15% of the total Aβ clearance in mice (Parodi-Rullan et al., 2020; Figure 3). Soluble LRPs (sLRPs) in plasma are derived from the cleavage of LRP by BACE1 (von Arnim et al., 2005). Approximately 70% of Aβ in the plasma directly binds to sLRPs, which have been identified as key components of the endogenous “sink” action that increases the clearance of peripheral Aβ and reduces the free levels in circulation, promoting the cell-surface LRP-mediated clearance of brain-derived Aβ across the BBB (Sagare et al., 2007). In the plasma of AD patients and AD transgenic mice, sLRP is oxidized and shows a decreased affinity with Aβ (Sagare et al., 2011; Figure 4).

## Amyloid-β Deposition Due to Blood–Brain Barrier Dysfunction

Experiments have demonstrated that BBB dysfunction results in the deposition of Aβ by increasing its production and preventing its normal transport through the BBB. Arguably, BBB dysfunction promotes Aβ production by activating BACE1 and γ-secretase (Ridler, 2018; Wang et al., 2018). The transport function of BBB is essential to maintain normal Aβ levels in the brain. Many receptors regulate Aβ transport in the BBB. The quantity and distribution of these receptors are affected by AD pathology, resulting in abnormal transport and deposition of Aβ. The effects of BBB damage on each receptor and its corresponding mechanism are discussed next.

### Low-Density Lipoprotein Receptor-Related Protein 1

The LDL receptor (LDLR) family member LRP1 is involved in various pathophysiologic processes, including Aβ clearance and its accumulation as a substance transporter and signal receptor (Shinohara et al., 2017; Bilousova et al., 2019). The LDL receptor (LDLR) family member LRP1 is involved in various pathophysiologic processes, including Aβ clearance and its accumulation as a substance transporter and signal receptor (Shinohara et al., 2017). LRP1 and its ligands have also been identified in senile plaques (Van Gool et al., 2019).

The expression of Aβ receptors alters with age and in AD patients. Shibata et al. reported that Aβ could be exported from the brain across the BBB through the LRP1, and identified a downregulation in LRP1 levels in the brain microvasculature of patients with AD (Shibata et al., 2000). Later experiments confirmed this finding (Gali et al., 2019). This led to the neurovascular hypothesis of AD, which proposes that defects in LRP1 lead to Aβ-efflux obstruction across the BBB and the subsequent accumulation of Aβ in the brain, which ultimately promote the progression of AD (Gali et al., 2019). The expression of LRP1 decreases with age as well as in patients with AD, as manifested in the whole brain and cerebral capillaries (Osgood et al., 2017). However, other experiments have led to different conclusions. In AD patients, LRP1 was found to be up-regulated in a cell-type-dependent manner (Devraj et al., 2016), which was manifested in the increased expression of LRP1 in neurons and astrocytes activated around senile plaques (Arelin et al., 2002). In addition, the affinity of LRP1 to Aβ_1__–__42_ was found to be higher than was the affinity to Aβ_1__–__40_ (Storck et al., 2016).

Low-density lipoprotein receptor-related protein 1 interacts with AβPP at the neuronal surface through its adaptor protein, Fe65, which enhances AβPP endocytosis and the generation of Aβ (Van Gool et al., 2019). Furthermore, LRP1 in neurons regulates Aβ cellular uptake and retention of Aβ in the brain (Shinohara et al., 2017).

Some studies found that repression of LRP1 leads to Aβ accumulation, eventually ameliorating cognitive deficits in mice, which supports the finding that LRP1 participates in the efflux of Aβ (Swaminathan et al., 2018). Several drugs promote Aβ clearance by increasing LRP1 expression in the brain or liver, including statins, pioglitazone, Withania somnifera, and the traditional Chinese medicine Linguizhugan decoction (Sehgal et al., 2012; Hu et al., 2018; Seok et al., 2019). In the physiological state, LRP1 regulates Aβ clearance from the brain through a three-step, continuous mechanism.

Low-density lipoprotein receptor-related protein 1, which is expressed in the endothelial cells and pericytes of the BBB, is a multifunctional scavenger receptor that mediates the clearance of Aβ from the brain through the BBB into the peripheral blood circulation (Zhou et al., 2015; Storck et al., 2018). LRP1 regulates the uptake and degradation of Aβ through neurons, astrocytes, and cerebrovascular smooth-muscle cells (Liu et al., 2017).

Soluble low-density lipoprotein receptor-related protein 1 (sLRP1) in the circulating plasma binds to >70% of free Aβ and acts as an additional aid. The Aβ and sLRP1 complex in the circulation is eliminated by the liver (Jiang et al., 2018). sLRP has been identified as a key part of the endogenous Aβ “sink” action in the plasma, which prevents circulating Aβ entering the brain and promotes sustained clearance (Seok et al., 2019). Nevertheless, sLRP is oxidized in patients with AD, which decreases the binding affinity for Aβ while increasing the free Aβ in the plasma, which may result in an increase in the amounts of Aβ entering the brain through RAGE (Deane et al., 2012; Seok et al., 2019).

**FIGURE 5:**
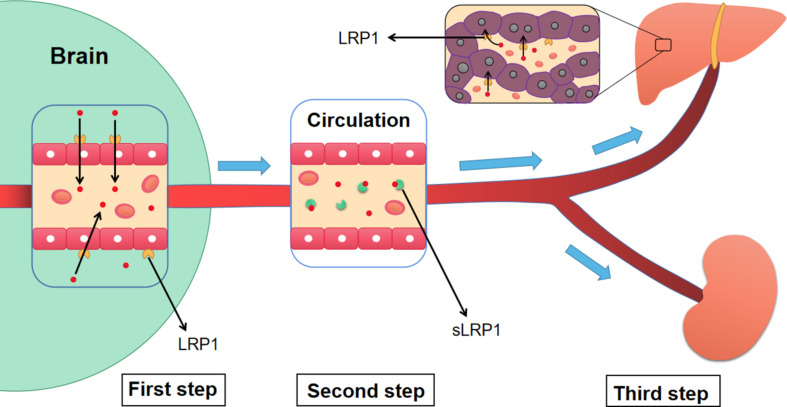
The three-step mechanism of LRP1 regulating the clearance of Aβ. Firstly, LRP1 expressed on the endothelial cells of BBB, promotes the transportation of Aβ from the brain to blood circulation. Then, sLRP1 in peripheral blood binds to free Aβ, which prevents Aβ from flowing into the brain and contributes to the continuous clearance of Aβ. Finally, LRP1 in hepatocytes promotes the clearance of circulating Aβ. Meanwhile, the sLRP1-Aβ complexes and free Aβ are also cleared by kidney. Aβ, Amyloid-β; LRP1, low-density lipoprotein receptor-related protein 1; sLRP1, soluble low-density lipoprotein receptor-related protein 1.

The liver is a source of Aβ. LRP1 on hepatocytes is related to the systemic clearance of circulating Aβ and affects Aβ metabolism in the brain (Seok et al., 2019). An increase in peripheral Aβ clearance reduces Aβ levels in rodent brains. In older rats, a decrease in LRP1 levels on hepatocytes and an associated decrease in circulating Aβ clearance were identified (Seok et al., 2019). Similar results were observed in aged squirrel monkeys (Mackic et al., 2002). Moreover, the kidney can also eliminate free Aβ and sLRP1-Aβ complexes (Sagare et al., 2007; Figure 5).

### Receptor for Advanced Glycation End Products

Receptor for advanced glycation end-products, which is a 35-kDa multiligand transmembrane receptor, is characterized by its ability to bind to AGEs as well as its involvement in neurologic dysfunction in AD patients (Wan et al., 2014). Neeper et al. (1992) first described RAGE as a member of the immunoglobulin (Ig) superfamily in 1992.

Receptor for advanced glycation end-products is expressed in various cell types, including neurons, vascular endothelial cells, vascular smooth-muscle cells, and gliocytes. The basal expression level of RAGE is low in a wide range of cell types while high expression levels can be detected in the lungs of healthy humans (Paudel et al., 2020). However, RAGE expression levels are upregulated in some pathological states, including atherosclerosis, diabetes, cancer, chronic inflammation, and chronic neurodegeneration (Hudson and Lippman, 2018).

Receptor for advanced glycation end-products binds a broad repertoire of molecules, including AGEs, Aβs, S100 calcium-binding protein B (S100B), macrophage-1 antigen (Mac-1), high-mobility group box 1 (HMGB1), products of non-enzymatic glycoxidation, and amphoterin (Saleh et al., 2013; Huang Y.Y. et al., 2020). The expression levels of RAGE are determined by the concentration of these ligands.

Receptor for advanced glycation end-products consists of intracellular, extracellular, and transmembrane domains. The extracellular domain is responsible for binding ligands (Koch et al., 2010). Subtypes include a soluble form of RAGE (sRAGE) and the full-length membrane-bound form (mRAGE). Compared with mRAGE, sRAGE lacks cytosolic and transmembrane domains (Paudel et al., 2020). sRAGE antagonizes the effects of the RAGE ligand by increasing cerebral blood flow and reducing inflammation (Xue et al., 2011). In addition, sRAGE hetero-oligomerizes with RAGE and binds to Aβ as a decoy receptor, thus reducing Aβ binding to mRAGE in the brain (Wang P. et al., 2016). These effects inhibit the formation of Aβ plaques and relieve neuroinflammation to delay AD progression. Furthermore, sRAGE expression is decreased in AD cases (Fuller et al., 2018).

Much evidence indicates that the neurotoxicity of Aβ in the brain is mediated by the activation of RAGE. Therefore, RAGE plays a key role in the progression of AD (Jarosz-Griffiths et al., 2016). Acting as a key transporter, RAGE interacts with Aβ and participates in its influx from the peripheral venous blood to the brain across the BBB, thereby promoting plaque formation in AD (Agrawal et al., 2018; Mantle and Lee, 2018). Evidence from numerous studies demonstrates that RAGE–ligand interactions activate multiple signaling pathways and their downstream events, which are associated with many chronic inflammatory diseases, including AD, stroke, diabetes, and arteriosclerosis (Somensi et al., 2017).

In AD, evidence shows that the combination of RAGE and Aβ lead to a series of reactions, including oxidative stress, reduced cerebral blood flow, and vascular dysfunction (Huang Y.Y. et al., 2020). Moreover, the binding of RAGE and Aβ results in the activation of microglia and the release of inflammatory factors, such as IL-1β, reactive oxygen species (ROS), tumor necrosis factor (TNF)-α, and plasminogen activator inhibitor-1 (PAI-1), which cause an inflammatory response and aggravate the disruption of brain homeostasis. In addition, Aβ–RAGE engagement results in sustained nuclear factor kappa-light-chain-enhancer of activated B cells (NF-κB) activation, which in turn increases RAGE expression and forms a positive-feedback loop causing inflammatory damage. A transient proinflammatory response is transformed into a chronic pathophysiological condition in the CNS, and the positive-feedback loop also damages endothelial cells and the BBB, exacerbating AD pathology (Batkulwar et al., 2018; Fang et al., 2018; Huang Y.Y. et al., 2020). Studies have also revealed a functional relationship between RAGE and TJ proteins and shown that Aβ_1__–__42_ destroys TJ proteins through a RAGE-dependent autophagy pathway (Chan et al., 2018).

Receptor for advanced glycation end-products is found in microglia, neurons, and astrocytes in the brains of patients with AD. In the physiological state, RAGE is expressed at low levels in the brain, whereas its expression in the endothelial cells, neurons, and microglia of patients with AD is significantly increased (Yang et al., 2020). RAGE mediates the neurotoxic effects of Aβ and increases Aβ expression. In addition, Aβ aggregation in the brain upregulates RAGE expression in transgenic AD models and in AD patients (Nan et al., 2019; Paudel et al., 2020). Multiple lines of evidence have shown that RAGE-antagonists block the binding of Aβ and RAGE and significantly reduce Aβ levels and plaque formation in AD models (Batkulwar et al., 2018).

### P-Glycoprotein

P-glycoprotein is a 140-kDa membrane protein and a member of the ATP-binding cassette (ABC) transporter superfamily (Chai et al., 2020b). It is expressed in the kidney, adrenal gland, gastrointestinal tract, liver, brain, lung, testis, eye, and skin (Wang et al., 2017). In the brain, P-gP is mainly expressed on the lumen (blood-facing) surface of the BBB endothelium, and helps limit the entry of brain-active drugs into the CNS using ATP (Chen et al., 2021). P-gP is also found in astrocytes, neurons, and microglia (Chai et al., 2020b; Vita et al., 2020). The expression and function of P-gP are compromised with age and in cases of AD (Guerreiro and Bras, 2015). Aβ is considered to be a P-gP substrate (Lam et al., 2001), and accumulating evidence supports its role in Aβ clearance from the brain (Jana et al., 2017).

The relationship between P-gP expression and Aβ deposition was explored for the first time in humans in 2002. Research revealed that the deregulation of P-gP expression induced Aβ deposition and the occurrence of cerebral amyloid angiopathy (CAA), which increased the possibility of developing AD (Rosas-Hernandez et al., 2020). Recent *in vivo* findings support the participation of P-gP in Aβ efflux from the brain in mice and humans (Chai et al., 2020a; Rosas-Hernandez et al., 2020). These data have been confirmed in a variety of cell lines, including mouse Lewis lung carcinoma (LLC1) cells, the immortalized human cerebral microvascular endothelial cell line hCMEC/D3, and human embryonic kidney 293 (HEK293) cells, supporting the suggestion that P-gP plays a role in Aβ efflux (Haran et al., 2019; Chai et al., 2020a).

Various experiments have explored the state of P-gP in AD. Decreased P-gP expression in AD results in impairment of Aβ clearance and there is a negative correlation between P-gP expression and Aβ_1__–__40_ plaques (Bourassa et al., 2019). In addition to P-gP expression levels, the functions of P-gP are also thought to be impaired in AD and aging (Hartz et al., 2018). The positron emission tomography (PET) tracer [11C] verapamil has been used to identify P-gP functions over the past two decades (Tournier et al., 2018; Zoufal et al., 2020). This approach has demonstrated reduced P-gP activity at the BBB in aging and neurodegenerative diseases (Bauer et al., 2019; Zoufal et al., 2020). In 2012, *in vivo* evidence was presented showing that BBB P-gP dysfunction occurs at both a regional and global level in AD patients (van Assema et al., 2012). Notably, several experiments reported that deposited Aβ reduces P-gP expression (Chai et al., 2020b). This inhibitory effect may be mediated by the RAGE–NF-κB or Wnt/β-catenin pathways (Park et al., 2014). In the early stages of AD, P-gP expression levels are up-regulated to compensate for and reduce Aβ accumulation. However, during the development of AD, Aβ deposition disrupts P-gP expression and function, eventually forming a vicious cycle intensifying the deposition of Aβ (Vogelgesang et al., 2002).

Quercetin increases the expression of P-gP in Caco-2 cells, chicken, mice, and humans (Bhutto et al., 2018; Pinheiro et al., 2020). One study demonstrated that ibuprofen treatment restores impaired levels of ABC sub-family B (MDR/TAP) member 1A (Abcb1a)/1b messenger RNA (mRNA) and P-gP expression in AD mice (Zhang et al., 2018). Ketone bodies (KBs) enhance the expression levels of LRP1 and P-gP, and the combined use of KBs (AcAc and βHB) improves Aβ transport through the BBB (Versele et al., 2020). Lack of P-gP leads to Aβ-clearance disorders and Aβ deposition. Extensive research has used different P-gP inhibitors, such as PSC833, XR9576, RU486, and RU49953, to reduce P-gP expression and found a reduction in related brain Aβ efflux and accumulation (Lam et al., 2001; Mistry et al., 2001). Knockout models further support this view. P-gP protein-coding genes in mice include Abcb1a and Abcb1b. Studies have shown Aβ accumulation in Abcb1a-knockout mice. Crossing Abcb1a-knockout mice with Tg2576 mice overexpressing AβPP resulted in significant Aβ-clearance inhibition. The Aβ-clearance ability of Tg2576 Abcb1a/b double-knockout mice was significantly lower than that of control mice (Wang W. et al., 2016). Moreover, many inducers of P-gP, including oleocanthal, bexarotene, PYR41, and rivastigmine, increase Aβ efflux and reduce Aβ deposition by increasing P-gP expression levels (Kuntz et al., 2015; Manda et al., 2016; Mohamed et al., 2016; Hartz et al., 2018). Recent experiments suggested that overexpression of P-gP increases Aβ clearance by inhibiting the expression of inflammatory factors (Zhang et al., 2018; Pan et al., 2020).

Notably, when P-gP is silenced, the expression of LRP1 in the brain is also significantly reduced. Therefore, it is difficult to determine whether impaired Aβ efflux is caused only by P-gP silencing. Other experiments also found that LRP1 deficiency was accompanied by a down-regulation in P-gP expression (Storck et al., 2018). Some studies suggest that the capacity of P-gP to extrude Aβ from the brain is weaker than that of LRP1, and that P-gP serves an auxiliary role to LRP1 in the transcytosis of Aβ. This process is divided into two steps: the first includes the transcytosis of Aβ from the brain into BMECs mediated by LRP1 at the abluminal surface; and the second includes the transcytosis of Aβ into the circulating blood regulated by P-gP at the luminal surface (Storck et al., 2016, 2018; Wang W. et al., 2016). However, some experiments have indicated that P-gP and LRP1 play a role in regulating Aβ efflux independently (Erickson et al., 2012).

In addition to these three transporters, there are others on the BBB that are related to Aβ transport including the neonatal Fc receptor (FcRn) and several members of the ABC family, such as ABCA1, ABCG4, and ABCG2 (BCRP), which have functions in transporting Aβ from the brain to the peripheral blood. Moreover, ABCA7 may inhibit the production of Aβ by affecting AβPP (Dib et al., 2021).

## Blood–Brain Barrier Impairment Caused by Amyloid-β Deposition

During the occurrence and progression of AD, the interaction of BBB dysfunction and Aβ deposition promotes the neurodegenerative process. This section discusses the mechanisms underlying this phenomenon.

One study revealed that immunizing Tg2576 AD mice with Aβ allowed the long-term restoration of damaged BBB (Dickstein et al., 2006). Another study showed that infusions with soluble Aβ compromised the BBB and caused cortical perivascular gliosis (Su et al., 1999). Further work has demonstrated that excessive Aβ generation and deposition increases BBB disruption, which plays a key role in the onset and development of AD (Montagne et al., 2017).

### Metalloproteinases (MMPs)

Activated MMPs degrade extracellular matrix proteins, TJ proteins, and basement membranes (Song et al., 2017), which are related to the development of many diseases, including cerebral infarction, atherosclerosis, multiple sclerosis, and AD (He J.T. et al., 2020). Significant increases in metalloproteinase 9 (MMP-9) have been observed in post-mortem AD tissues (Asahina et al., 2001). Aβ treatment of human vascular smooth-muscle cells (VSMCs) increases mRNA expression of membrane type 1 (MT1) MMP and subsequently actives matrix metalloproteinase 2 MMP-2 (Jung et al., 2003). In BMECs associated with AD there is increased expression of MMP-2 and MMP-9, minimal expression of TJ-associated proteins like CLDN-1 and CLDN-5, and significantly increased BBB permeability (Hartz et al., 2012). Silencing MMP genes improved the permeability of the BBB (Hu et al., 2009). The expression of MMPs is related to increased permeability of the BBB. GM 6001, which is a broad-spectrum MMP inhibitor, partially reverses the inhibition of endothelial cells induced by Aβ (Wan et al., 2015). When MMP-2 and MMP-9 expression levels are increased and ZO-1 expression levels are decreased, occludin levels also decrease. At the same time, an increase in BBB permeability has been observed (Zhang et al., 2012). In addition, the interaction of Aβ-RAGE promoted the expression of MMP-2 and MMP-9, decreased the expression of TJ-related proteins, and increased BBB permeability (Hartz et al., 2012), Several substances that block the interaction of Aβ-RAGE can inhibit the expression of MMPs (Du et al., 2012). For example, the polyclonal antibody of RAGE inhibits the up-regulation of MMP-2 and MMP-9, and alters protein induced by Aβ by blocking the function of RAGE (Wan et al., 2015). The effects of Aβ treatment are effectively inhibited by transiently knocking down RAGE (Wan et al., 2015).

### Reactive Oxygen Species (ROS)

Reactive oxygen species are natural by-products of cell metabolism that lead to lipid peroxidation, activation of apoptosis and damage to local tissues (Sole et al., 2019). Therefore, ROS play a leading role in many aspects of neurodegenerative diseases (Cockerill et al., 2018). Both microdialysis administration of Aβ_1__–__40_ and intracerebroventricular infusion of Aβ_1__–__42_ in rats increase levels of ROS. The latter is achieved by reducing important endogenous antioxidant enzymes such as glutathione-S-transferase, mitochondrial magnesium-superoxide dismutase 2 (SOD2), and glutathione peroxidase (Kim et al., 2003). In addition, ROS induce the phosphorylation of TJ proteins (CLDN-5, occludin, and ZO-1), which triggers the destruction of BBB integrity; this process is mediated by the up-regulation of protein tyrosine kinase (PTK) along with diminished protein tyrosine phosphatase (Cockerill et al., 2018). Under normal physiological conditions, sLRP increases the clearance of peripheral Aβ and reduces the free Aβ in the circulation, whereas sLRPs are oxidized in the plasma of AD patients (Sagare et al., 2007). This suggests that high levels of ROS may damage the function of proteins that play a significant role in neurovascular mechanisms.

### Nuclear Factor-κB (NF-κB)

After entering the brain through the BBB, Aβ deposition can activate NF-κB, promote the secretion of proinflammatory cytokines and lead to the occurrence of neuroinflammation and BBB destruction (Deane et al., 2003). The damaged BBB loses its normal transport function and is unable to transport substances necessary for the nervous system, such as nutrients, electrolytes and vitamins. Finally, a positive-feedback pathway leads to the continuous activation of NF-κB and a chronic pathological state (Maczurek et al., 2008; Origlia et al., 2009). The accumulation of Aβ induces activation of microglia and subsequent release of pro-inflammatory molecules (Heneka et al., 2015). Microglia are in a “rest or quiescent state” under physiological conditions. Under pathological conditions, microglia are activated with the turn on of phagocytic activity and the release of pro-inflammatory cytokines (Arcuri et al., 2017). In addition, it has been suggested that microglia are the first cells to degrade soluble and fibrillar Aβ aggregates through receptor interactions. This process may lead to the production and activation of a variety of toxic molecules that ultimately affect the function of BBB (Moore et al., 2002). Specifically, the interaction of Aβ with a variety of receptors, including CR3 (Mac-1) fAβ and Aβ/SRA, will trigger increased expression of NF-κB, and secretion of ROS, TNF-α, complement components and other pro-inflammatory substances (Yang et al., 2011; Zhang et al., 2011; Soto-Rojas et al., 2021b). In the transgenic AD mouse model, it was observed that the accumulation of Aβ caused NF-κB upregulation with, the destruction of BBB integrity, and a decline in mouse learning ability (Scheffer et al., 2021). Another study showed that NF-κB signaling mediates the inhibition on P-gp expression induced by Aβ_1__–__42_, thereby affecting the function of BBB (Zamani et al., 2020). Another study showed that NF-κB signaling mediates the reduction of P-gp expression induced by Aβ_1__–__42_, thereby affecting the function of BBB (Park et al., 2014). Besides, the breakdown of BBB also depends on the activation of the Aβ-RAGE-NF-κB signaling pathway (Chen et al., 2018). It is reasonable that controlling the activation of microglia can inhibit the production of NF-κB, protect BBB, and ameliorate neurological impairments (Xie et al., 2018).

### Ca^2+^-Calcineurin (CaN)

Ca^2+^ is closely related to the formation of various cell connections, and maintains the TJs structure and BBB integrity (Wan et al., 2014). Aβ oligomers induce calcium influx in the neural cells (Kook et al., 2013; Arbel-Ornath et al., 2017), The elevated cytosolic calcium could lead to reduced expression of ZO-1 and other TJ proteins in the plasma membrane thus induces increased BBB permeability (Kook et al., 2012). CaN, a serine/threonine protein phosphatase, is a heterodimer composed of a 60-kDa catalytic A subunit (CaN-A) and a 19-kDa regulatory B subunit (CaN-B) (Rusnak and Mertz, 2000; Dos et al., 2020). Ca^2+^ activation of CaN occurs via CaM activation or via calpain, which cleaves out the auto-inhibitory domain (Wu et al., 2007). Some recent experiments employed FK506 to suppress the up-regulation of CaN caused by Aβ. The results showed that FK506 inhibited Aβ-induced changes on the expression of TJs and the integrity of BBB. These findings support the opinion that Aβ-induced TJs destruction is mediated by the intracellular Ca^2+^-CaN signaling pathway (Kook et al., 2013; O’Neal et al., 2018; Tapella et al., 2018). In addition, disturbance of calcium homeostasis is an important cause of neurotoxicity in AD (Popugaeva et al., 2017). In the animal model, synaptic toxicity occurs around Aβ plaques, which is believed to be related to the chronic activation of CaN (Hopp et al., 2018). The pharmacological inhibitory treatment on CaN could also block the synaptotoxicity of Aβ (Hopp et al., 2018).

Current knowledge of the specific mechanisms of Aβ damage to the BBB is insufficient and further exploration is required.

In addition to the association between Aβ and BBB mentioned in this review, chronic neural inflammation is also a remarkable hypothesis of the pathogenesis of AD, as inflammation could be the primary cellular stressor for elderly people. Both gene editing and gene expression regulation have great potential to modulate the progression of AD. The pathological factors that connect type 2 diabetes and AD, as well as that are involved in the development of AD after traumatic brain injury, hold promising research prospects. Despite the numerous clinical investigations in-progress, exploring diagnostic biomarkers for AD has always been a conundrum. Since BBB damage occurs in AD brain, many substances including miRNA and protein will enter the peripheral blood, and these molecules may include relevant markers of BBB damage. In addition, numerous studies have identified that neuron-derived exosome secreted after AD will carry pathological proteins and other tissue damage factors into the peripheral blood. For this reason, detection of BBB-related injury markers in peripheral blood exosome of AD patients may open a new avenue for early diagnosis of AD. Lastly, the use of traditional medicine with BBB protective effects, such as Linguizhugan, may become a future research direction in AD treatment.

## Conclusion

The interaction between Aβ and the BBB affects the progression of AD. Aβ is not only related to neurotoxicity and neuronal loss, but can also destroy BMECs and TJ related proteins in the BBB, ultimately destroying the integrity and function of the BBB. In addition, a large number of experiments revealed that BBB dysfunction promotes Aβ production and accelerates the deposition of Aβ in the brain. In general, BBB dysfunction and Aβ deposition results in a vicious cycle, which together lead to the occurrence and development of AD. AD is a complex disease requiring additional investigation regarding its pathogenesis and treatment.

## Author Contributions

DW, PL, and FC conceived, designed, and drafted the manuscript. DW wrote the original draft preparation. ZH, ZY, and XG contributed to the review and edit of the manuscript. XG contributed to the language modification and guidance. All authors read and approved the final manuscript.

## Conflict of Interest

The authors declare that the research was conducted in the absence of any commercial or financial relationships that could be construed as a potential conflict of interest.
